# Time Course of Muscle Damage and Inflammatory Responses to Resistance Training with Eccentric Overload in Trained Individuals

**DOI:** 10.1155/2013/204942

**Published:** 2013-01-22

**Authors:** Bernardo Neme Ide, Lázaro Alessandro Soares Nunes, René Brenzikofer, Denise Vaz Macedo

**Affiliations:** ^1^Laboratory of Exercise Biochemistry, Biochemistry Department, Biology Institute, State University of Campinas, 13083-970 Campinas, SP, Brazil; ^2^Sport Science Department, Faculty of Physical Education, Metropolitan College of Campinas (Metrocamp), 13035-270 Campinas, SP, Brazil; ^3^Faculty of Physical Education, Sport Science Department, State University of Campinas, 13083-851 Campinas, SP, Brazil

## Abstract

The purpose of this study was to observe the time course of muscle damage and inflammatory responses following an eccentric overload resistance-training (EO) program. 3 females (23.8 ± 2.6 years; 70.9 ± 12.7 kg; 1.6 ± 0.08 m) and 5 males (23.8 ± 2.6 years; 75.1 ± 11.2 kg; 1.8 ± 0.1 m) underwent thirteen training sessions (4 × 8–10 eccentric-only repetitions—80% of eccentric 1RM, one-minute rest, 2x week^−1^, during 7 weeks, for three exercises). Blood samples were collected prior to (Pre) and after two (P2), seven (P7), nine (P9), eleven (P11), and thirteen (P13) sessions, always 96 hours after last session. The reference change values (RCV) analysis was employed for comparing the responses, and the percentual differences between the serial results were calculated for each subject and compared with RCV_95%_. Four subjects presented significant changes for creatine kinase at P2, and another two at P13; six for C-reactive protein at P2, and three at P11; two for neutrophils at P2, P4, and P13, respectively; and only one for white blood cells at P2, P4, P7, and P9, for lymphocyte at P7, P9, and P13, and for platelet at P4. We conclude that EO induced high magnitude of muscle damage and inflammatory responses in the initial phase of the program with subsequent attenuation.

## 1. Introduction

Resistance-training protocols with eccentric overload (EO) have been investigated about their efficiency on strength and muscle cross-sectional area increases [1–4]. Experimental observations reported suggest that this training variation might be superior for both strength and hypertrophy development when compared to conventional concentric/eccentric training protocols [1–3]. EO is imposed once the maximal voluntary force is greater in this kind of muscle action [5, 6], thus promoting a relatively lower workload for the eccentric phase during the conventional programs.

Meanwhile, the high-intensity unaccustomed eccentric component is characterized by inducing greater muscle damage incidence with significant acute decreases in muscle function [7], acute-phase inflammatory responses [8–12], and increases in plasmatic activity of myofibrillar proteins such as creatine kinase, lactate dehydrogenase, and myoglobin [12–15]. Instead, there have also been reports that a bout of eccentric exercise at several weeks interval results in a marked reduction in the symptoms associated with muscle damage [9]. This adaptation of eccentric exercise-inducing protection against subsequent tissue damages has been referred as repeated bout effect [16].

Observing the data on the initial drop in performance induced by the eccentric activity [7, 15, 17], recent researches have aimed at a very important aspect of the physical training process: the monitoring of biochemical and immunological markers and also the performance [18–20]. High training loads with insufficient recovery periods have been suggested to induce overreaching and overtraining in team sport players [18, 21], and the monitoring of these responses along with the training period may be critical for the identification of such events.

Hematological and biochemical analyses are often used to identify fatigue and recovery in athletes during the training seasons [18], although relatively few studies have systematically examined the use of EO resistance-training protocols for monitoring such factors [2–4, 12]. To compare individual blood parameter values with reference intervals obtained from a physically active population may also be a useful tool to monitor the adaptive effects of exercise. However, this form of comparative analysis has certain limitations, as laboratory results may be influenced by biological variation [22], hindering the clinical results interpretation, particularly those from consecutive analysis performed for the same individual. 

Considering this aspect to compare the serial results, in the present study, we adopted the reference change values (RCV) or critical difference analysis. The RCV is a tool employed to verify if the difference between two consecutive samples is significant and biologically relevant, considering the various components of intrinsic variation affecting laboratory assays. These components include factors related to laboratory activity (preanalytical and analytical variation) and those related to normal intraindividual biological variation [23]. As such, the RCV defines the percentage of change that should be exceeded when considering the analytical and biological variation inherent to a test, in order to evaluate significant changes between two consecutive measurements. 

Bearing this in mind, the aim of this study was to observe the time course of muscle damage and inflammatory responses to resistance-training with EO adopting the RCV to compare the serial results. Our initial hypothesis was to observe high magnitude of muscle damages on the initial phase of the program, together with greater inflammatory responses. However, a subsequent attenuation of these events by the repeated bout-effect occurrence was expected [9, 16].

## 2. Methods

### 2.1. Experimental Design

Thirteen training sessions were performed twice a week, performed at the same hour of the day, and under the supervision of the researchers involved. One familiarization session with the resistance exercise equipments happened three weeks before the program started. Blood samples were collected in eight distinct time points throughout the program and always before the individuals started the sessions. These time points were Pre (prior to the first session), P2 (after two sessions), P7 (after seven sessions), P9 (after nine sessions), P11 (after eleven sessions), and 96 hours after the last training session (P13). 

### 2.2. Participants

Eight healthy subjects (3 female: age: 23.8 ± 2.6 years; body mass: 70.9 ± 12.7 kg; height: 1.6 ± 0.08 m; % body fat: 29.6 ± 4.3; and 5 male: age: 23.8 ± 2.6 years; body mass: 75.1 ± 11.2 kg; height: 1.8 ± 0.1 m; % body fat: 20.0 ± 4.9) participated in the study. The inclusion criteria included being engaged in resistance-training programs for at least one year, and no intake of exogenous anabolic-androgenic steroids, drugs, medication, or dietary supplements with potential effects on physical performance is recorded. Training programs usually performed by the subjects consisted of 3–5 sets of 6–12 repetitions with 1-2-minute rest interval between sets, performed 4-5 times per week. Subjects gave written informed consent after being advised about the purposes and the risks associated with the study. Subjects fasted for one hour prior to the blood collection, being also required to refrain from strenuous exercise and the consumption of alcohol, tobacco or caffeine 48 hours before the testing sessions. During the period of the study, none of the participants reported any kind of infection state that could affect his/her immune responses despite the inflammation induced by the exercise. Research Ethics Committee (019/2004) approved the experimental protocol.

### 2.3. Strength Tests

The one repetition maximal test (1RM) was employed a fortnight prior to the beginning of the study, aiming the prescription of the resistance-training intensity (% 1RM). Since the present study makes a distinction between the concentric and eccentric phase of the movement, it was necessary to employ a specific test to access the maximum weight to be bore by the eccentric-only muscle action. The typical 1RM test reflects only the maximum weight that can be lifted using a concentric action—designated as 1RMcon. This specific evaluation is referred as 1RM eccentric (1RMecc) [24], and it is adopted due to the observations that skeletal muscles are capable of developing much higher forces when they contract eccentrically compared to concentrically [24].

The 1RMcon testing was conducted using the methods described by Brow and Weir [25], and for the 1RMecc the methods described by Hollander and coworkers [24]. There were three-to-five single trials for the validation of the test, which was the subject using proper form and completing the entire lift in a controlled manner without assistance [26].

### 2.4. Training Program

The program consisted of thirteen sessions over seven weeks, performed on Tuesdays and Thursdays, always at the same time of the day and under the supervision of the researchers involved. Prior to each session, subjects completed a standardized warm up program of static stretching exercises, and a specific warm up of 8 repetitions with approximately 50% of the estimated 1RMcon for each exercise. Training protocol consisted of 4 sets of 8–10 eccentric-only repetitions with 80% of 1RMecc, and one minute of rest between sets. The concentric phase of the movement was performed with the assistance of the researchers, and the exercises employed were Bench Press, 45-degree Leg Press, and Bent-Over Rows.

### 2.5. Blood Samples

Blood samples were collected under standardized conditions: 2.0 mL of total venous blood was collected in vacuum tubes containing EDTA/K3 to determine hematological parameters, and 8.0 mL of venous blood was collected in tubes with a Vacuette (Greiner Bio-one) gel separator in order to obtain serum for biochemical measurements. Blood samples were collected 96 hours after the last training bout, in the morning after 12 hours of fasting, in a seated position, transported at 4°C to the laboratory within 30 minutes, centrifuged under refrigeration at 1.800 ×g for 10 minutes, immediately separated, and protected from light.

### 2.6. Blood Analysis

Hematological analyses were conducted using a KX-21N (Sysmex, Brazil) automated hematology analyzer. The analysis included red blood cell count (RBC), hemoglobin concentration (Hb), hematocrit (Ht), mean corpuscular volume (MCV), mean corpuscular hemoglobin (MCH), mean corpuscular hemoglobin concentration (MCHC), erythrocyte distribution width (RDW), white blood cell (WBC) count, lymphocyte (LYNF) count, neutrophil (NEUTR) count, and platelet count (PLT).

Biochemical measurements were conducted with commercial kits (Wiener Lab; Rosario, Argentina) and with an Autolab (Boehringer, Mannheim, Germany) analyzer. The assay included creatine kinase (CK) activity, and C-reactive protein (CRP). To minimize analytical variations, the same technician tested all samples without changing reagent lots, standards, or control materials.

### 2.7. Statistical Analysis

The percentual differences between the serial results were calculated for each subject and compared with RCV_95%_ to detect significant changes [27]. The RCV_95%_ to detect significant changes was based on the following formula: RCV_95%_ = (2)^1/2^∗Zp(CV_*A*_^2^ + CV_*I*_^2^)^1/2^  where  2^1/2^  is to verify difference between 2 time points, Zp : Z score (probability 95% = 1.96),  CV_*A*_: analytical coefficient of variation, and CV_1_: intraindividual coefficient of variation [28]. The RCV_95%_ for physically active subjects employed in this study was previously determined [23]. The RCV_95%_ considered were RBC = 8.3%; Hb = 8.0%; Ht = 8%; MCV = 2.3%; MCH = 2.8%; MCHC = 3.3%; RDW = 6.1%; WBC = 43.9%; LYNF = 40.5%; NEUTR = 65.3%; PLT = 21.5%; CK = 119.3%; CRP = 206%. Significant change for each analyte was considered when its percentage change in two subsequent measurements exceeded the determined RCV_95%_. The reference intervals were based on results published by our laboratory based on a physically active population: RBC (4.4–5.6 10^12^/L), Hb (13.0–16.1 g/dL), Ht (39.5–48.0%), MCV (80.9–94.9 fL), MCH (26.1–31.6 pg), RDW (12.1–14.3%), WBC (4.5–10.1 10^9^/L), LYNF (1.2–3.3 10^9^/L), NEUTR (1.8–6.7 10^9^/L), PLT (140–337 10^9^/L), CK (<1309 U/L), and CRP (<19.8 mg/L) [29]. Pearson's coefficient of correlations was used in order to establish relationships between the analysis. Statistical comparisons were performed using GraphPad Prism version 5.00 for Windows (GraphPad Software, San Diego, CA, USA).

## 3. Results

Table 1 shows significant changes in CK and CRP accessed throughout the training period. CK and CRP presented significant changes at specific time points, but not for all subjects. Four subjects presented significant changes in CK activity at P2 (+1719%, +1250%, +1281%, and +312% resp.), and other two at P13 (+391% and +139%, resp.). For CRP six subjects presented significant changes at P2 (+1100%, +243%, +3800%, +2500%, +1400%, and +2400%, resp.), one at P4 (+567%), other at P9 (+3200%), three at P11 (+300%, +3400%, and +3900%, resp.), and other at P13 (+1500). Pearson's coefficient of correlation at P2 for CK and CPR (*R* = 0.31), CK and NEUTR (*R* = −0.24), and CK and WBC (*R* = −0.09) was not significant (*P* > 0.05). Table 1 presents the significant changes in CK and CRP throughout the training period and the specific subjects who presented these alterations.

Only two subjects presented significant changes for NEUTR (Table 2). Subject 7 presented changes at P2 (+90%) and P4 (−71%), and subject 6 at P13 only (+60%). In addition, subject 7 also presented significant changes for WBC at P2 (+53%), P4 (−57%), P7 (+71%), and P9 (+49%), for LYNF P7 (+344%), P9 (+57%), and P13 (−44%), and for PLT at P4 (−25%). No significant changes were observed for RBC, PLT, Ht, MCH, MCHC, RDW, MCV, and Hb throughout the training period. Table 2 presents the significant changes in the aforementioned markers and the specific subjects presenting alterations.

## 4. Discussion

Our main findings were that four subjects presented significant changes for CK at P2, and other two at P13. For CRP six subjects presented significant changes at P2, one at P4, other at P9, three at P11, and other at P13. Only two subjects presented significant changes for NEUTR. Subject 7 presented changes at P2 and P4, and subject 6 at P13 only. In addition, subject 7 also presented significant changes for WBC at P2, P4, P7 and P9, for LYNF at P7, P9, and P13, and for PLT at P4. No significant changes were observed for RBC, PLT, Ht, MCH, MCHC, RDW, MCV, and Hb. No significant correlations were observed between CK and CRP as well as WBC and NEUTR at P2.

To the best of our knowledge, this is the first study to employ the RCV specifically for physically active subjects when comparing the temporal behavior of inflammatory and muscle damage responses to EO. Individual blood analyses provided by the RCV allowed the identification of the specific subjects seeming to be more susceptible for inflammatory processes and muscle damage. Conventional statistical analysis considers the probability of occurrence of one event for a group and not for specific subjects, what may mask or disregard the more responsive individuals. These results appear to be very important for future studies aiming at monitoring these events.

CK responses presented by four subjects at P2 supported our initial hypothesis to observe high magnitudes of muscle damage on the initial phase of the program. The subsequent attenuation of this event confirmed the occurrence of the repeated bout effect, with the exception of two subjects with significant increases of CK at P13 in relation to RCV, but within the RI for physically active subjects (Table 1). The repeated bout effect refers to the adaptation whereby a single bout of eccentric exercise protects against muscle damage from subsequent bouts [16]. The potential adaptations that explain the phenomena have been categorized as neural, mechanical, and cellular. Regarding the cellular adaptations there is evidence of longitudinal addition of sarcomeres and adaptations in the inflammatory response following an initial bout of eccentric exercise, limiting also the proliferation of damage.

Despite four individuals not presenting significant increases in CK at P2, we cannot completely state that they did not experience muscle injuries. Evidences in the literature report that CK plasma activity is not considered as a gold-standard evaluation of muscle damage because it does not present a significant linear correlation with muscle functions and ultrastructural changes in muscle following exercise [17, 30, 31]. Presently, muscle functions measurements are considered the most indicated methods for quantifying injuries because the event results in an immediate and prolonged reduction in these parameters, persisting over the entire span of the progression of the degenerative and regenerative processes [17, 32]. On the other hand, plasma activities of myofibril proteins are not evidenced over this entire time course of degenerative and regenerative processes, and even when evidenced, they may or may not be correlated with the magnitude of the functional decrements [17, 32]. We recognize that one limitation of the present study was not having a quantification of muscle functions parallel to plasma CK activity in order to establish possible correlations between them. CRP responses do not correlate to CK, emphasizing that elevated CRP levels may be associated to damage in nonskeletal muscle tissue.

Similar to CK, CRP responses were characterized by two time points when subjects appeared to express higher responses. Despite the similarity of response at P2, no significant correlations with CK were found, confirming the observations made by previous studies [13]. Literature emphasizes that elevated CRP levels may simply mark the clearance of modified molecules or necrotic tissue in an effort to limit damage associated with the inflammatory process [33]. Providing a valid prediction of the progression of vascular lesions in the absence of acute infection, the increases in CRP levels may be associated to damage in nonskeletal muscle tissue [33], thus, explaining the poor correlation between CK and CRP responses found. It is important to observe that all subjects presented significant changes in relation to RCV but within the traditional RI for physically active subjects (Table 1).

Time course of muscle damage and inflammatory responses have also been investigated in two recent studies [34, 35]. The first study evaluated the process following an international rugby union game. An acute-phase inflammatory response reflected through immediate increases in serum cortisol and IL-6, followed by delayed increases in serum CK (14 hours) activity and CRP (38 hours) was observed. The findings suggested that a rugby match elicits disturbances in host immunity, which last up to 38 hours into the recovery period. The second research observed the process together with performance changes, following a soccer match (in the morning of the game day, immediately after, and 24, 48, 72, 96, 120, and 144 hours after match). Performance deteriorated 1-to-4 days after match, an acute-phase inflammatory response consisting of a postmatch peak of leukocyte count, 24-hour peak of CRP, and 48-hour peak of CK were observed.

According to the behaviors reported in the aforementioned studies, it becomes evident that the moment for collection of blood samples is a crucial aspect for their observation (i.e., the inflammatory process appears to have an acute-phase response with a subsequent attenuation). The moment of blood collection in the present study was 96 hours after the last training bout, which could not be the most adequate one for the observation of the time course for some markers. These observations may help us to explain the reasons why only two subjects presented significant changes for NEUTR at P13, and only one for WBC (at P2, P4, P7, and P9), LYNF (at P7, P9, and P13), and PLT (at P4). Other subjects may have expressed an early-stage (24–48 hours after last bout) acute-phase inflammatory responses, which was not detected 96 hours after the last bout.

Specific resistance-training inflammatory responses were reported by Simonson and Jackson [11]. Blood samples were drawn at before, after, 15 minutes after, and 30 minutes after exercise. All leukocyte subpopulations, except for basophils and eosinophils, increased at after exercise but the counts declined at after, 15, and 30 minutes after exercise. Only NEUTR did not return to preexercise levels by 30 minutes after exercise. The majority of resistance exercise-induced leukocytosis was due to an increase in circulating LYNF and monocytes. In the study, the authors suggested that by the lack of large alterations and rapid recovery from cell number, resistance training is not immunosuppressive. Meanwhile, we highlight that the data of the aforementioned studies were not analyzed according to the RCV for physically active subjects.

## 5. Conclusions

EO resistance training protocol induced high magnitudes of muscle damage and CRP responses on the initial phase of the program, with a subsequent attenuation of the event. Such behavior confirms the occurrence of the repeated bout effect for this particular training protocol, except for two subjects. Nevertheless, CRP responses do not correlate to CK, emphasizing that elevated CRP levels may be associated to damage in nonskeletal muscle tissue.

## Figures and Tables

**Table 1:** Significant changes in CK and CRP and the specific subjects presenting alterations. RI: reference interval for physically active subjects [23]; Δ: difference between two consecutive analyses.

Analyte	Moment	Subject	Measured values: latter − former (Δ)	% change
CK RCV_95%_ = 119.3% RI < 1309 U/L	P2	3	2455 − 135 (2320)	+1719
	P2	4	1417 − 105 (1312)	+1250
	P2	5	2278 − 165 (2113)	+1281
	P2	7	465 − 113 (352)	+312
	P13	1	339 − 69 (270)	+391
	P13	8	404 − 169 (235)	+139

CRP RCV_95%_ = 206% RI < 19.9 mg/L	P2	1	1.2 − 0.1 (1.1)	+1100
	P2	3	2.4 − 0.7 (1.7)	+243
	P2	5	3.9 − 0.1 (3.8)	+3800
	P2	6	2.6 − 0.1 (2.5)	+2500
	P2	7	1.5 − 0.1 (1.4)	+1400
	P2	8	2.5 − 0.1 (2.4)	+2400
	P4	4	2 − 0.3 (1.7)	+567
	P9	2	3.3 − 0.1 (3.2)	+3200
	P11	1	0.4 − 0.1 (0.3)	+300
	P11	3	3.5 − 0.1 (3.4)	+3400
	P11	7	4 − 0.1 (3.9)	+3900
	P13	8	1.6 − 0.1 (1.5)	+1500

**Table 2:** Significant changes in NEUTR, WBC, LYNF, and PLT and the specific subjects presenting alterations. RI: reference interval for physically active subjects [23]; Δ: difference between two consecutive analysis.

Analyte	Moment	Subject	Measured values: latter − former (Δ)	% change
NEUTR RCV_95%_ = 65.3% RI = 1.8–6.7 (10^9^ cel/L)	P2	7	9.4 − 4.9 (4.5)	+90
	P4	7	2.7 − 9.4 (−6.7)	−71
	P13	6	6.2 − 3.9 (2.3)	+60

WBC RCV_95%_ = 43.9% RI = 4.5–10.1 (10^9^ cel/L)	P2	7	12.2 − 8.0 (4.2)	+53
	P4	7	5.2 − 2.2 (3.0)	−57
	P7	7	8.9 − 5.2 (3.7)	+71
	P9	7	13.3 − 8.9 (4.4)	+49

LYNF RCV_95%_ = 40.5% RI = 1.2–3.3 (10^9^ cel/L)	P7	7	5.6 − 1.3 (4.3)	+344
	P9	7	8.8 − 5.6 (3.2)	+57
	P13	7	7.0 − 3.9 (3.1)	−44

PLT RCV_95%_ = 21.5% RI = 140–337 (10^9^ cel/L)	P4	7	234 − 176 (58)	−25
